# Supplementary material for: Isolation, Identification and Pollution Prevention of Bacteria and Fungi during the Tissue Culture of Dwarf Hygro (Hygrophila polysperma) Explants
Source: Microorganisms. 2022 Dec 15;10(12):2476. doi: 10.3390/microorganisms10122476 (PMC9785981; doi:10.3390/microorganisms10122476)
Supplement: Supplementary file 1 [file microorganisms-10-02476-s001.zip › microorganisms-2037376-supplementary.pdf]

Supplementary Information (SI)

Figure legends

Figure S1 The inhibition effects of the antibiotics for bacteria in LB broth. The bacteria were subjected to LB broth containing 100 µg/mL of ampicillin (Amp), 50 µg/mL of kanamycin (Kan), 250 µg/mL of erythromycin (Ery) and 200 µg/mL of spectinomycin (Spc) for 9 days, respectively. The bacterial growth was observed and took photos. A-L indicated the bacterial strains *K. oryzendophytica* (*Ko*), *Pantoea* BH (BH), *B. amyloliquefaciens* BV2007 (BV), *B. siamensis* (*Bsi*), *B. amyloliquefaciens* Y5 (Y5), *B. koreensis* (*Bk*), *B. aryabhattai* B8 (B8), *Ensifer* BO (BO), *B. zanthoxyli* (*Bz*), *E. cloacae* (*Ec*), *Enterobacter* Glu2 (Glu2) and *Enterobacter* E24 (E24), respectively.

Figure S2 The inhibition effects of chloramphenicol for bacteria in LB broth. The bacteria were grown in LB broth containing 25 µg/mL of chloramphenicol (Chl) for 10 days, and the bacterial growth was observed and took photos. The bacterial strains *Ko*, BH, BV, *Bsi*, *B. subtilis* (*Bsu*), *Bk*, B8, BO, *Bz*, *Ec*, *K. michiganensis* (*Km*), Glu2 and E24, respectively.

Figure S3 The inhibition effects of three antifungal agents for fungi in PDA plates. Three fungi were grown in PDA plates (Control) or the PDA plates added with potassium sorbate (PS), sodium benzoate (SB), and sodium diacetate (SD) at different percentage concentration of 0.0125% (w/v), 0.25% (w/v), 0.5% (w/v) and 1.0% (w/v), respectively.

Figure S4 The influence of two kinds of antibiotics on *H. polysperma* explants during tissue culture. *H. polysperma* plantlets were grown in MS medium supplemented with two antibiotics at series concentration of 50 µg/mL of Kan and 25 µg/mL of Chl (Kan+Chl), 1/2 (Kan+Chl), 1/5 (Kan+Chl) and 1/10 (Kan+Chl) were tested, respectively.

Figure S5 The influence of three antifungal agents on *H. polysperma* explants during tissue culture. *H. polysperma* plantlets were grown in MS media supplemented with PS, SB, and SD, respectively. The fungal inhibition capability was tested for two months at the percentage concentration of 0.015625% (w/v), 0.03125%(w/v), 0.0625% (w/v), 0.1% (w/v), 0.2% (w/v) and 0.5% (w/v), respectively. The appropriate percentage concentration of PS indicated by the red arrow.

Figure S6 The normal growth of *H. polysperma* explants treated with a mixture of three antimicrobial agents in MS media. *H. polysperma* stem explants (A) and (C) in MS media without the antimicrobial agents, emerged serious pollution, indicated by red arrows. *H. polysperma* explants (B) and (D) had good growth performance in modified MS media, supplemented with 5 µg/mL of Chl, 10 µg/mL of Kan and 0.015625% PS.

Figure S1

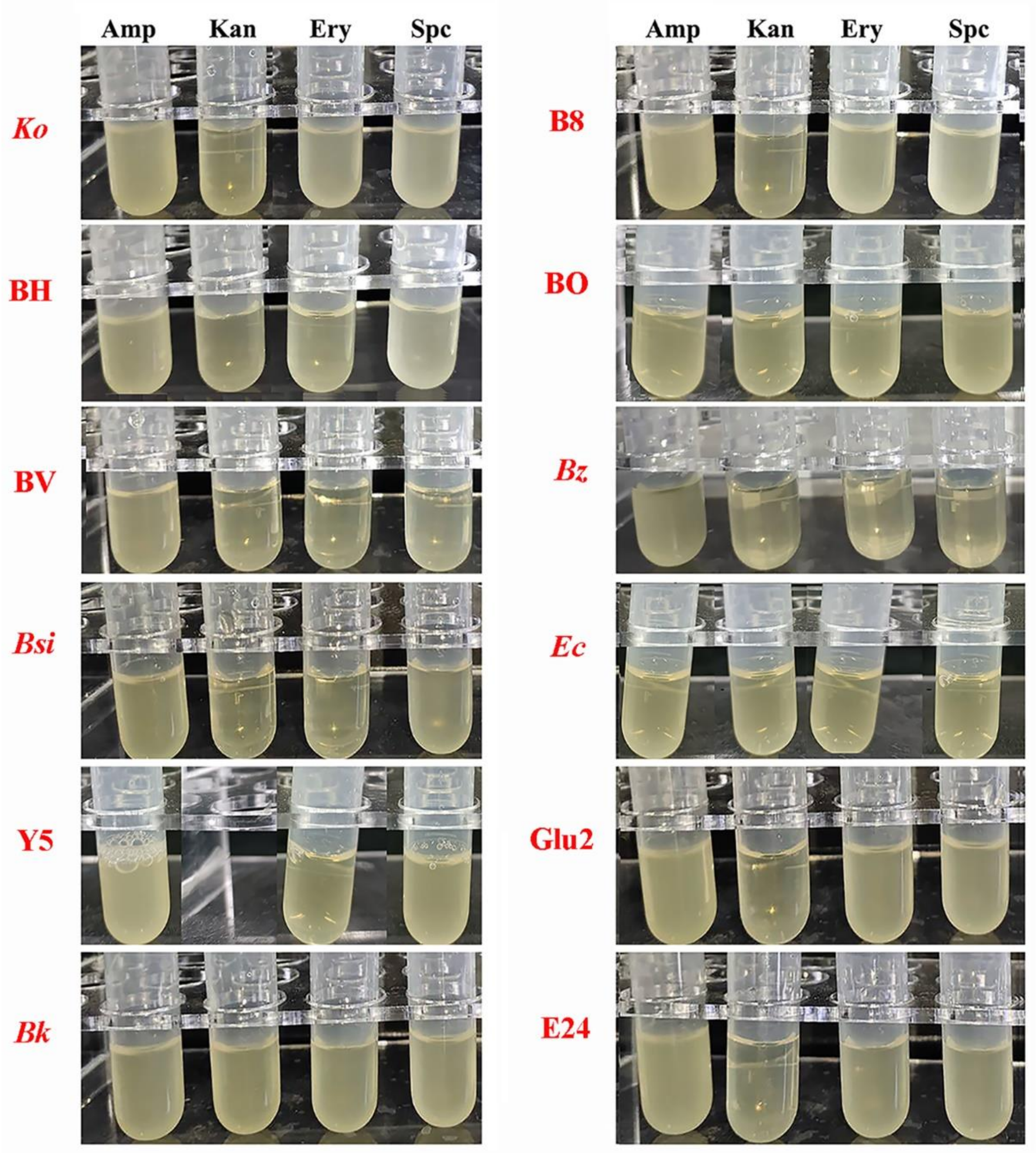
Amp
Kan
Ery
Spc
Amp
Kan
Ery
Spc
Ko
BH
BV
Bsi
Y5
Bk
B8
BO
Bz
Ec
Glu2
E24

Figure S2

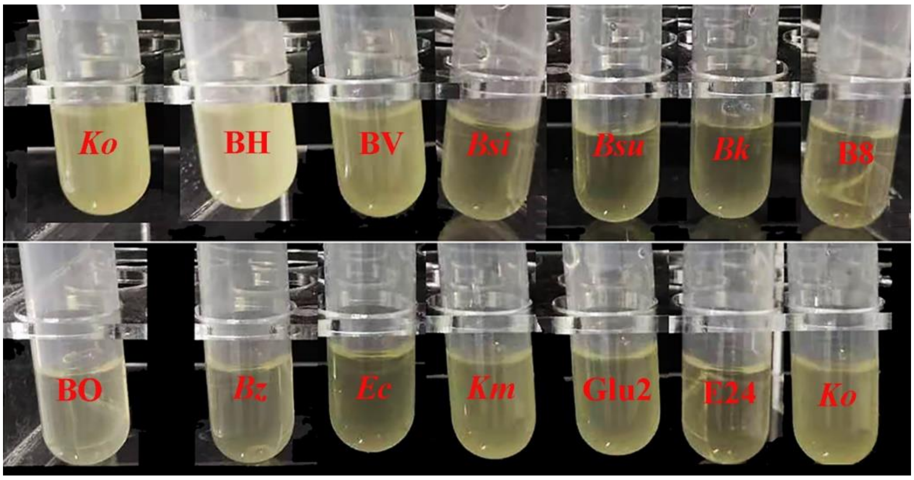

Figure S3

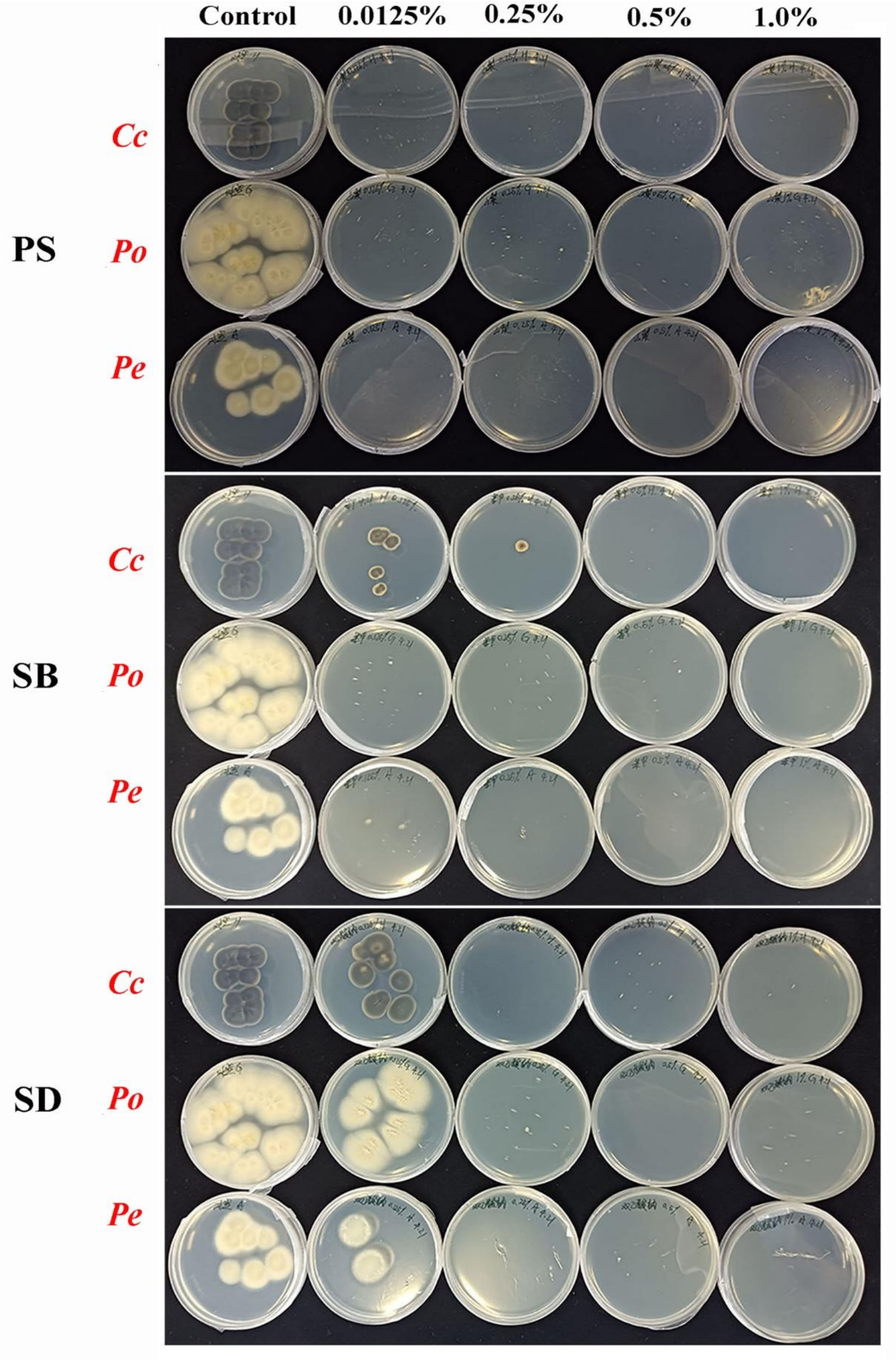

Figure S4

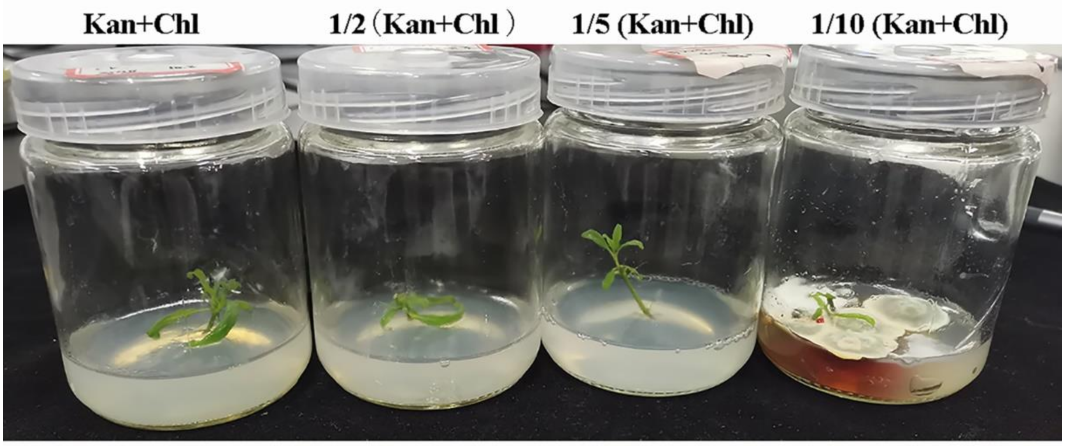

Figure S5

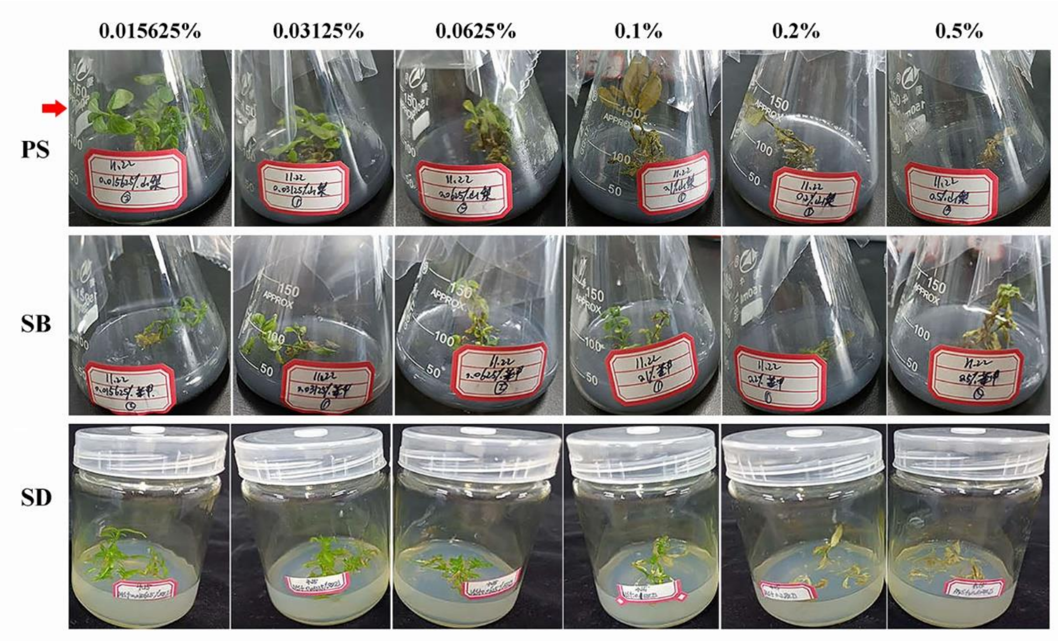

Figure S6

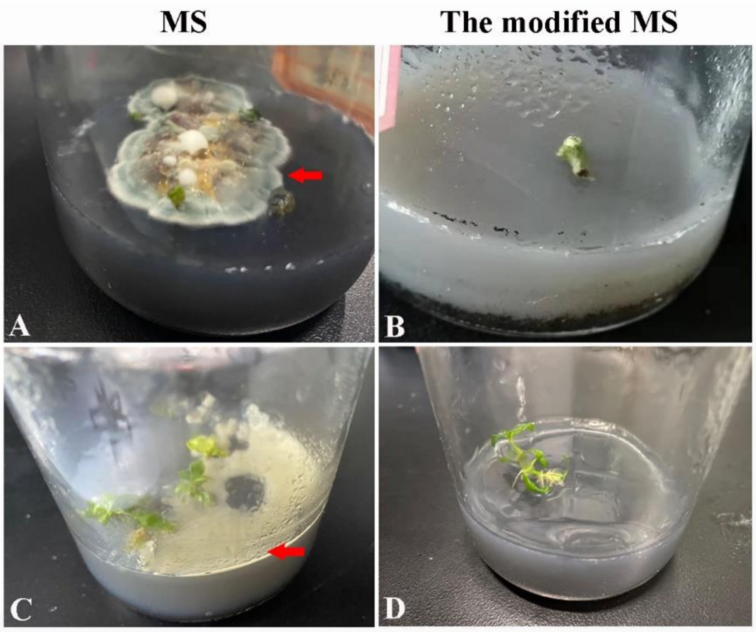

Table S1 Phenotypes of explants or seedlings in MS media treated with three antifungal agents.

**Antifungal agents**	**Times of treatment**	**Concentrations of preservatives**					
		**0.015625%**	**0.03125%**	**0.0625%**	**0.1%**	**0.2%**	**0.5%**
Potassium sorbate (PS)	7 d	Normal	Normal	Normal	Wilt	Serious wilt	Death
	14 d	Normal	Normal	Wilt	Death	Death	Death and rotten
Sodium benzoate (SB)	7 d	Normal	Normal	Normal	Wilt	Death	Death
	14 d	50% pollution	Wilt	Wilt	Serious wilt	Death	Death
Sodium diacetate (SD)	7 d	Normal	Normal	Wilt	Wilt	Death	Death
	14 d	Wilt	Wilt	Wilt	Serious wilt	Death	Death
